# BAMClipper: removing primers from alignments to minimize false-negative mutations in amplicon next-generation sequencing

**DOI:** 10.1038/s41598-017-01703-6

**Published:** 2017-05-08

**Authors:** Chun Hang Au, Dona N. Ho, Ava Kwong, Tsun Leung Chan, Edmond S. K. Ma

**Affiliations:** 1 Division of Molecular Pathology, Department of Pathology, Hong Kong Sanatorium & Hospital, Happy Valley, Hong Kong SAR, China; 2 Department of Surgery, The University of Hong Kong, Happy Valley, Hong Kong SAR, China; 3 Department of Surgery and Cancer Genetics Centre, Hong Kong Sanatorium & Hospital, Happy Valley, Hong Kong SAR, China; 4 Hong Kong Hereditary Breast Cancer Family Registry, Happy Valley, Hong Kong SAR, China

## Abstract

Amplicon-based next-generation sequencing (NGS) has been widely adopted for genetic variation detection in human and other organisms. Conventional data analysis paradigm includes primer trimming before read mapping. Here we introduce BAMClipper that removes primer sequences after mapping original sequencing reads by soft-clipping SAM/BAM alignments. Mutation detection accuracy was affected by the choice of primer handling approach based on real NGS datasets of 7 human peripheral blood or breast cancer tissue samples with known *BRCA1*/*BRCA2* mutations and >130000 simulated NGS datasets with unique mutations. BAMClipper approach detected a *BRCA1* deletion (c.1620_1636del) that was otherwise missed due to edge effect. Simulation showed high false-negative rate when primers were perfectly trimmed as in conventional practice. Among the other 6 samples, variant allele frequencies of 5 *BRCA1*/*BRCA2* mutations (indel or single-nucleotide variants) were diluted by apparently wild-type primer sequences from an overlapping amplicon (17 to 82% under-estimation). BAMClipper was robust in both situations and all 7 mutations were detected. When compared with Cutadapt, BAMClipper was faster and maintained equally high primer removal effectiveness. BAMClipper is implemented in Perl and is available under an open source MIT license at https://github.com/tommyau/bamclipper.

## Introduction

**Figure 1:**
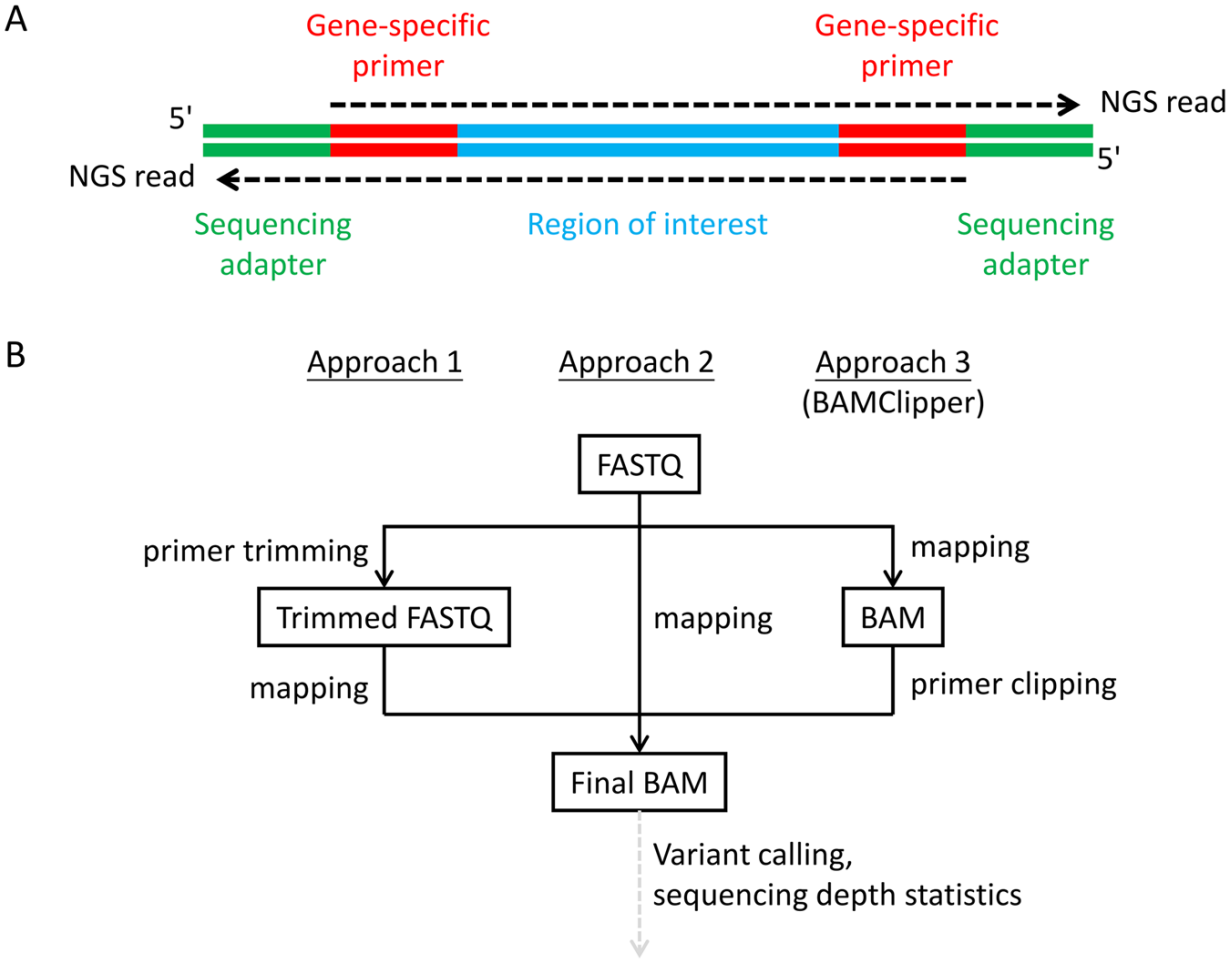
Amplicon library design and bioinformatics approaches of handling gene-specific primers. (**A**) Gene-specific primer sequences are present as part of NGS reads. The observed primer sequences are usually identical to reference genome sequence but may be slightly different due to errors in primer synthesis and/or sequencing. Common read mapping tools are not aware of the amplicon library design and thus map the primers as if they are part of region of interest that lies in between. Although sequencing adapters may exist as part of NGS reads (depending of amplicon length and sequencing read length), they will become soft-clipped after mapping due to the lack of similarity to reference genome by design. Soft-clipped part of alignments is usually ignored by downstream processing. (**B**) In primer handling approach 1, primer sequence was trimmed from sequencing reads (in FASTQ format) and the shorter trimmed reads are mapped to give BAM alignments for downstream variant calling and quality control such as sequencing depth statistics. In approach 2, original reads are directly mapped that primers are present in BAM alignments as if they are part of region of interest. In approach 3 represented by BAMClipper, reads are also directly mapped but BAM alignments are further processed to soft-clip primer sequences as if they were sequencing adapters.

Amplicon-based next-generation sequencing (NGS) has been one of the major platforms for high-throughput germline^1, 2^ and somatic mutation detection^3–7^. Various experimental strategies^8^, including traditional or multiplex polymerase chain reaction (PCR) and selective circularization probes, all converge to a common molecular design of NGS sequencing library (Fig. 1A).

The NGS reads usually contain both gene-specific primers (from the reagent) and region of interest (ROI, from DNA sample of interest) and have fixed start positions defined by primer sites. Common read mapping tools map primer sequences in the same way as ROI because of their equally high similarity to reference genome sequence. Common bioinformatics workflows may thus include a primer trimming step before mapping to remove primer sequence from NGS reads and avoid potential interference (approach 1, Fig. 1B), for example variant allele frequency (VAF) dilution by apparently wild-type primer sequences. However, variant calling edge effect occurred for single-nucleotide variants (SNV) such that SNV in ROI near primer sites were prone to escape variant calling^9^.

In some specific situations, primer trimming is not necessary (approach 2, Fig. 1B). Some genes of clinical importance have well-known hotspot mutational profile^10^ (e.g. gain-of-function mutations of *EGFR* and *KRAS*) and primers are designed to avoid any potential edge effect. In case of short exons or ROI, amplicon tiling is not needed so that ROI of one amplicon is not overlapped with and not interfered by primer of another amplicon. However, there are genes having diverse mutational profile^10^ (e.g. loss-of-function mutations of *BRCA1* and *BRCA2*) or less characterized profile and full exons are usually covered by tiling amplicons. For gene panels having these 2 kinds of genes, both approaches 1 and 2 are needed to complement each other for comprehensive mutation detection. Mutation detection accuracy is maximized, but computing and manual interpretation efforts are doubled.

In this study, we devised primer clipping after mapping as an alternative approach of primer removal (approach 3, Fig. 1B). Existing tools of approach 3 include PcrClipReads (https://github.com/lindenb/jvarkit/wiki/PcrClipReads) that performs primer clipping on non-overlapping amplicons by design. GATK^11^ ClipReads performs primer clipping based on exact sequence matching regardless of its position within sequencing reads. Thus, GATK ClipReads will improperly handle overlapping amplicons due to sequence cross-matching and cannot recognize primers in case of any mismatch (sequencing errors for example). MiSeq Reporter Custom Amplicon Workflow (https://www.illumina.com/systems/miseq/software/miseq-reporter.html) properly clips primers from overlapping amplicons but the software is proprietary and supports specific sequencing platforms and commercial amplicon designs only. We implemented a tool called BAMClipper, which natively supports overlapping amplicons and any gene panel design. During evaluation of a breast and ovarian cancer gene panel for peripheral blood and breast cancer tumour tissue, BAMClipper approach detected *BRCA1* and *BRCA2* mutations that could otherwise be missed in conventional approaches due to edge or dilution effect. By experiments and simulation, insertion or deletions (indels) were shown to be susceptible to variant calling edge effect as in SNV. In addition to improving mutation detection accuracy, BAMClipper was more efficient in computation and maintained equally high primer removal effectiveness when compared with conventional primer trimming approach.

## Results

### Variant calling edge effect when primers are trimmed

**Figure 2:**
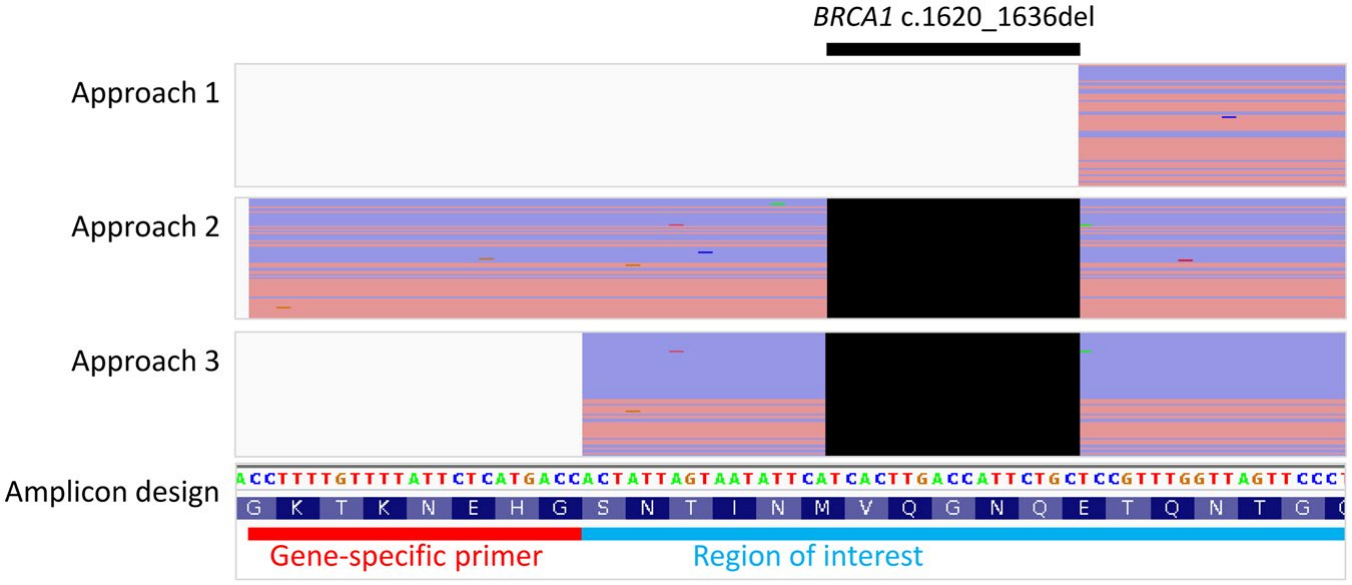
A *BRCA1* deletion escaped from variant calling when primers were trimmed before mapping. NGS read alignments of *BRCA1* c.1620_1636del allele from three primer handling approaches are shown in conjunction with the amplicon design and reference genome sequence. Individual forward and reverse sequencing reads after any soft-clipping were represented by red and purple horizontal lines, respectively. The expected deletion event (black box) was present in the alignments from approaches 2 and 3 only.

During evaluation of an amplicon-based breast and ovarian cancer gene panel with positive controls, we encountered a false-negative result for a *BRCA1* 17 nt deletion (c.1620_1636del). BWA-MEM alignments of the corresponding ROI were found to be soft-clipped when gene-specific primers were trimmed from NGS sequencing reads before read mapping (approach 1, Fig. 2). The soft-clipping removed wild-type sequence adjacent to the deletion site from read alignments of deletion allele. Alignment pileup therefore did not contain any deletion events for conventional variant callers to report. When primer trimming was skipped (approach 2) or primer clipping was performed after mapping (approach 3), the deletion was properly aligned and detected by variant callers (Fig. 2). These observations remained the same when the alignment algorithm was replaced with BowTie 2 (see Supplementary Fig. S1). Since SNV were known to be susceptible to variant calling edge effects in amplicon sequencing^9^, we hypothesized that indels were also susceptible to similar edge effects.

**Figure 3:**
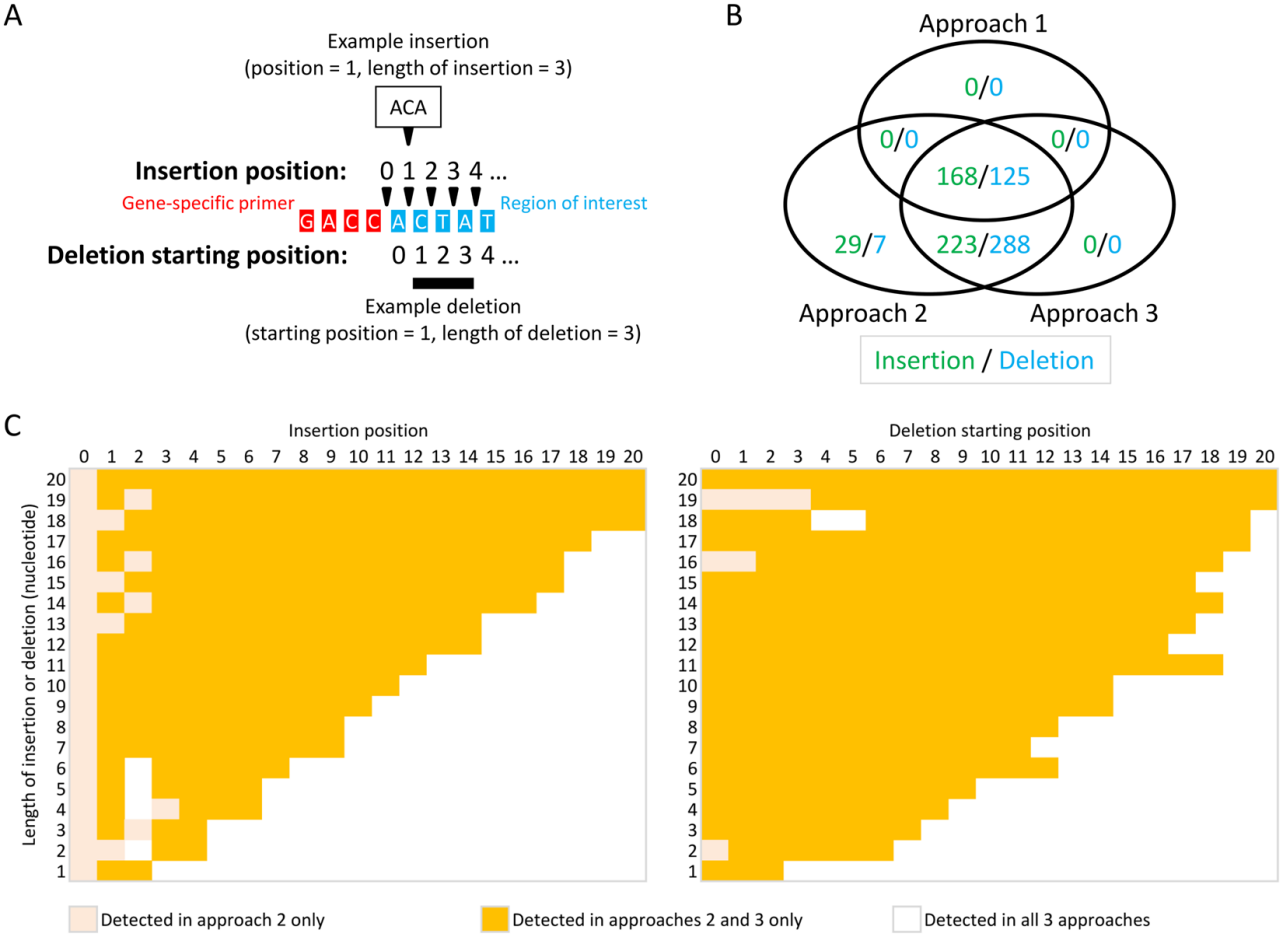
Indels are susceptible to variant calling edge effects as shown by simulation. (**A**) Simulation scheme of 420 insertions and 420 deletions with 20 different lengths at 21 different positions. (**B**) Venn diagram of called insertions or deletions in 3 approaches of primer handling. (**C**) Heat map of length and position of called insertions or deletions.

We simulated NGS reads representing 420 unique insertions and 420 unique deletions of different lengths (1 to 20 nt) at 21 different positions of the aforementioned *BRCA1* amplicon (Fig. 3A). All three approaches of primer handling were applied to the simulated reads for variant calling. In approaches 1, 2 and 3, 40%, 100% and 93% of insertions were detected and 30%, 100%, 98% of deletions were detected, respectively (Fig. 3B). If primers were trimmed from sequencing reads before mapping, indels were susceptible to variant calling edge effects. Longer indels and indels closer to gene-specific primer boundary were more susceptible to the edge effects (Fig. 3C). Regarding the 29 insertions (7%) and 7 deletions (2%) missed in approach 3, although BAMClipper clipped the primer sequences properly and the corresponding indels remained in both alignments and alignment pileup, they were not called by variant caller SAMtools.

To explore whether the edge effects also applied to overlapping amplicons, we simulated NGS reads for 68045 unique insertions and 64637 unique deletions similarly (length 1 to 20 nt) at 3446 different positions for the entire *BRCA1* exon 11. A total of 38 overlapping amplicons covered the region. In approaches 1, 2 and 3, 88.0%, 96.7% and 96.5% of insertions were detected and 76.9%, 96.7%, 93.2% of deletions were detected, respectively (see Supplementary Fig. S2). Similar edge effect was observed even in overlapping amplicon setting.

### VAF dilution effect when primers are not removed

**Figure 4:**
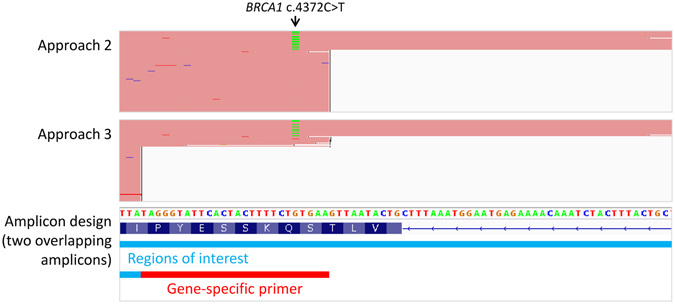
Dilution of variant allele frequency when primers are not clipped after mapping. NGS read alignments in *BRCA1* c.4372C>T region from primer handling approaches 2 and 3 are shown in conjunction with the amplicon design and reference genome sequence. The c.4372C>T mutation is located in the region of interest of one amplicon and gene-specific primer site of another amplicon. Since primer sequences retained in approach 2 contributed to wild-type allele frequency, VAF of c.4372C>T was in turn underestimated by 82% (9% in approach 2 and 51% in approach 3).

Since higher indel detection rate was demonstrated in approaches 2 and 3, we evaluated these approaches with 6 additional *BRCA1*/*BRCA2* positive controls (Table 1). Although all 6 mutations were detected in both approaches 2 and 3, the variant allele frequencies (VAF) of approach 2 (range: 9–60%) were consistently lower than approach 3 (range: 46–74%). Five of the 6 mutations were found to overlap with a gene-specific primer of another overlapping amplicon owing to the tiling amplicon design (Fig. 4). Primer sequences remained in approach 2 contributed to wild-type allele frequency and in turn underestimated VAF within region of interest of other overlapping amplicons. VAF underestimation ranged from 17% to 82% (Fig. 4) and VAF was not affected by BAMClipper if the mutation did not overlap with other primer site (Table 1). Since approach 3 properly handled primer sequences in NGS reads, downstream VAF determination was not interfered by overlapping primer sites. Overall, although approach 2 was robust to detect indels near primer, it was susceptible to VAF dilution effect. Approach 3 was versatile to detect indels near primer and mutations overlapping with other primer sequences.

**Table 1:** Variant allele frequency underestimation due to overlapping primer site.

Sample	Mutation	VAF		Overlap with other primer site?	VAF underestimation
		Approach 2	Approach 3		
NDH1	*BRCA1* c.4372C>T	9%	51%	Yes	82%
PMH1	*BRCA2* c.8023A>G	19%	49%	Yes	61%
TWH1	*BRCA2* c.1261C>T	50%	74%	Yes	32%
TWH2	*BRCA2* c.3109C>T	51%	51%	No	0%
TWH3	*BRCA1* c.502A>T	17%	46%	Yes	63%
QMH1	*BRCA1* c.3858_3861del	60%	72%	Yes	17%

### Effective primer removal and efficient computing

Performance characteristics of BAMClipper were further evaluated by various scenarios of primer clipping. Cutadapt (a representative tool of approach 1) was also applied to the same scenarios for benchmarking. BAMClipper removed 99.96% of gene-specific primers without affecting the regions of interest (i.e. without over-clipping), achieving comparable primer removal efficiency as Cutadapt (99.82% of gene-specific primers removed) (see Supplementary Fig. S3). For genes with well-known hotspot mutation profile, mutation detection sensitivity of approach 3 was as good as approaches 1 and 2 (see Supplementary Table S3), with one notable difference: the 52 nt hotspot deletion in *CALR* was detected in approaches 2 and 3 but not approach 1 due to similar edge effect. BAMClipper was consistently faster than Cutadapt, particularly for larger amplicon panel, longer sequencing reads and more sequencing reads (see Supplementary Fig. S3).

## Discussion

Amplicon-based next-generation sequencing has been widely adopted for genomic DNA mutation detection in both clinical diagnostics and research settings. Common bioinformatics workflows utilize tools originally designed for random fragment sequencing library (e.g. whole-genome sequencing, exome sequencing by hybridization capture). Although the tools are largely compatible with amplicon library, bioinformatics challenges exist in some scenarios, such as the detection of *FLT3* internal tandem duplication in acute myeloid leukaemia by amplicon sequencing^3^. In this study, we presented the bioinformatics challenges encountered during evaluation of a breast and ovarian cancer gene panel, namely variant calling edge effect and variant allele frequency dilution effect when handling gene-specific primers in amplicon sequencing library.

Edge effect led to a false-negative result of a *BRCA1* 17 nt deletion if primer trimming was performed according to common bioinformatics workflows. We further showed that such edge effect was not specific to this particular mutation but generally applicable to both single amplicon setting (65.1% false-negative among 840 unique indels) and overlapping amplicon setting (17.4% false-negative among 132682 unique indels). Indels were thus shown to be susceptible to similar edge effect reported for single-nucleotide variants^9^.

When primer trimming was skipped for a panel with amplicon tiling, evaluation of 6 additional *BRCA1*/*BRCA2* mutations revealed that 5 of the mutations were susceptible to VAF dilution effect (under-estimation by 17% to 82%). The dilution effect applied to both SNV and indels. Since the evaluated breast and ovarian cancer panel used amplicon tiling to cover protein-coding regions, 31% of ROI are overlapping with primer of other amplicon(s) and susceptible to the dilution effect. Whether a mutation affected by dilution effect would be missed by variant calling might depend on the variant calling algorithms (e.g. any VAF threshold) and sequencing depth uniformity among overlapping amplicons. Even though the 6 tumour DNA samples were derived from patients with a germline mutation (VAF >30% expected), VAF of a mutation dropped to below 10%. The situation represented possible false-negative scenarios of two common primer handling approaches in germline and somatic mutation detection due to the two effects.

Suitability of primer trimming depends on the genes of interest. For genes with well-known hotspot mutational profile (e.g. gain-of-function mutations of *EGFR* and *KRAS*) and their hotspot regions well covered by primers without amplicon tiling, primer trimming is not mandatory. However, for genes with diverse mutational profile (e.g. loss-of-function mutations of *BRCA1* and *BRCA2*) that necessitated their whole coding regions to be covered by amplicon tiling, primer trimming avoided dilution effect at the expense of sensitivity of mutations near primers due to edge effect. Taking the evaluated breast and ovarian cancer panel as an example, 40 nt of region of interest (20 nt of each end) for each amplicon was susceptible to edge effects if indels up to 20 nt are targeted. Since the mean ROI length is 110 nt for this panel to accommodate somatic mutation detection from degraded DNA (e.g. formalin-fixed paraffin-embedded tissue), 36% of ROI are susceptible to edge effect. When a single panel comprise both kinds of genes or genes with less characterized mutational profile, both primer handling approaches were needed to complement each other. We demonstrated that primer clipping was a general approach for mutation detection in both genes with diverse mutational profile and genes with well-known hotspot mutational profile (Fig. 2 and Supplementary Table S1). Although some commercial gene panel products implemented proprietary primer clipping solution to handle both kinds of genes properly (e.g. MiSeq Reporter results of TruSight Myeloid panel did not need redundant primer clipping by BAMClipper), there was a lack of general bioinformatics tool for primer clipping of most other gene panel designs.

We implemented BAMClipper as a novel approach of primer handling. By clipping primers after mapping, mutation detection accuracy was improved by minimizing false-negative results caused by edge effect and dilution effect. Well-established variant calling and quality control tools (e.g. SAMtools and bam-readcount) were tested to be compatible with this approach. Although the junction of primer and ROI was shown to be a variant calling blind spot by simulation of a single amplicon (even though the primers were properly clipped), the junction was covered by another amplicon in the evaluated panel that ROI overlapped by at least 1 nt. Since amplicon tiling was a common practice in commercial or custom panel design, few existing panels were actually affected.

The choice of primer handling approaches was shown to affect germline and somatic mutation detection accuracy. False-negative *BRCA1*/*BRCA2* mutation could affect the management of a high-risk cancer patient and his/her family members and could result in a missed opportunity of targeted therapy (e.g. poly(ADP-ribose) polymerases inhibitors in breast and ovarian cancers^12^).

Since BAMClipper was implemented in Perl and did not involve new file format, it could be deployed in existing bioinformatics workflows with minimal efforts. It was computation-efficient because the primers were already aligned by mapping tools and BAMClipper manipulated the alignments. Computing time saved by mapping shorter trimmed reads in conventional approach was usually exceeded by the trimming process. Even though advances in trimming algorithms could result in perfect trimming with minimal computing time, edge effect could persist as shown in the simulation. BAMClipper maintained the same high level of primer removal effectiveness as conventional trimming approach. We expected the new primer handling approach could improve amplicon-based germline and somatic mutation detection workflows.

To conclude, primers should be removed after mapping original sequencing reads for optimized genetic variation detection by amplicon sequencing. BAMClipper is an open-source, fast and effective tool to remove primers from mapped alignments of amplicon sequencing reads.

## Methods

### Implementation of BAMClipper algorithm

The BAMClipper algorithm was implemented mainly in Perl to read mapped alignments (standard SAM/BAM format^13^) and corresponding primer design (standard BEDPE format^14^) and return modified alignments in the same format. Gene-specific primer sequences were identified by comparing mapped positions of input alignments and reference positions of primer design (no de novo sequence alignment process was involved and thus the algorithm tolerated sequencing errors in observed primer sequences). Input alignments (a series of Concise Idiosyncratic Gapped Alignment Report or CIGAR operators in the alignment field of SAM/BAM format) were modified so that primer sequences were represented as S operators instead of M, I or D operators, as if they were sequencing adapters not matching to the reference genome and hence became soft-clipped during mapping. Output primer-clipped alignments remained in the standard BAM format that no modification of downstream variant calling (e.g. SAMtools^13^ and GATK^11^) or quality control (e.g. coverage-depth analysis by bam-readcount) was needed for analysis.

BAMClipper was tested to be sequencing platform-independent by clipping primers from amplicon sequencing reads from both Illumina MiSeq and Ion Torrent PGM platforms. It was shown to be aligner-independent by processing BWA-MEM and BowTie 2 alignments of same sequencing data. It was tested on CentOS Linux 5.5 and depended on the built-in Perl 5 installation of any Linux/Unix-like environment without compilation. A multi-threaded pipeline was also implemented (a single shell script bamclipper.sh) to leverage multiple processor cores and required SAMtools^13^ and GNU Parallel (https://www.gnu.org/software/parallel/).

### Germline and somatic mutation detection by a breast and ovarian cancer panel

Totally 7 positive control DNA samples were employed to evaluate the Qiagen GeneRead DNAseq v2 Human Breast Cancer Panel, which comprised 2915 amplicons targeting 44 genes. DNA was extracted from 1 peripheral blood sample and 6 frozen tumour tissue samples of 7 breast cancer patients with a known germline *BRCA1*/*BRCA2* mutation recruited from Hong Kong Hereditary and High Risk Breast Cancer Programme and samples obtained from the tissue bank of Hong Kong Hereditary Breast Cancer Family Registry^1^. PCR amplicons were prepared according to manufacturer’s instructions followed by sequencing library preparation based on KAPA Library Preparation Kit for Illumina platforms version 1.13. Paired-end sequencing runs were performed on a MiSeq with reagent kit v3 (2 × 230 nucleotides (nt)). Primer sequences were trimmed from sequencing reads by Cutadapt^15^ version 1.4.1 (parameters -m 0 -e 0.1 -n 2). Original sequencing reads or primer-trimmed reads were mapped to reference genome sequence using BWA-MEM^16^ version 0.7.7 (default parameters) or BowTie 2^17^ version 2.2.9 (default parameters). Variant calling and variant allele frequency (VAF) measurement was performed by SAMtools^13^ version 1.3 and assisted by manual alignment examination using IGV^18^ version 2.1.30.

### Simulation of sequencing reads for insertions and deletions

*In silico* simulation targeted the single *BRCA1* amplicon from the breast and ovarian cancer panel covering the *BRCA1* mutation c.1620_1636del (amplicon: chr17:41245872-41246030; ROI excluding both gene-specific primers chr17:41245895-41246009). A single pair of sequencing reads was simulated for each unique insertion or deletion with 0% error rate and 100% VAF. Insertion sequence was based on a template without known similarity to reference genome (5′-ACACTCTTTCCCTACACGACGCTCTTCCGATCT-3′) and 3′ trimmed to desirable length. Simulated reads of 840 unique indels were mapped to reference genome by BWA-MEM version 0.7.7 (default parameters) followed by variant calling using SAMtools version 1.3.

Another similar simulation was performed for the entire *BRCA1* exon 11 (chr17:41243442-41246887) in the same breast and ovariant cancer panel. Sequencing reads of all 38 overlapping amplicons were simulated for each unique insertion or deletion with 0% error rate and 100% VAF. Indels were excluded for simulation if they overlapped with primer binding site and sequencing reads could not represent the mutations.

### Performance characterization

Performance of BAMClipper and Cutadapt was characterized using amplicon NGS datasets of a myeloid neoplasm gene panel with 568 amplicons (Illumina TruSight Myeloid sequencing panel). Data based on peripheral blood sample of a de-identified healthy adult with normal complete blood profile^3^ was used to assess computing time with variable amplicon count, sequencing read length and sequencing read count, while data of the HapMap sample NA12878 (sample 2 of the public datasets “MiSeq v3: TruSight Myeloid (Coriell & HorizonDx, Pool 1)” available from https://basespace.illumina.com) was used to assess primer removal effectiveness. Data based on 8 mutation control samples from a previous study^3^ were used to compare the mutation detection sensitivity of all 3 primer handling approaches. Identical Cutadapt parameters as in the previous Methods were employed to work on FASTQ sequencing reads. Default BAMClipper parameters were used to work on corresponding BWA-MEM alignments (default parameters with BWA-MEM computing time ignored). Wall-clock computing time of BAMClipper and Cutadapt was measured based on 16 CPU cores (Intel Xeon E5 processors) (see Supplementary Note).

For primer removal effectiveness, BWA-MEM alignment depth of original sequencing reads (2 × 151 nt) of ROI or gene-specific primers was measured by bam-readcount (https://github.com/genome/bam-readcount) and served as reference. Reference positions of depth <500X were excluded from the following analysis. Alignment depth of BAMClipper and Cutadapt final BAM files was also measured similarly to calculate median decrease of depth after primer removal for each amplicon. Data from each amplicon were handled separately so that depth measurement was not interfered by any overlapping amplicons.

For amplicon count, one million (1 M) read pairs (2 × 275 nt) were first randomly sampled. Full panel (568 amplicons, 5 replicates) or random sample of 50 to 550 amplicons (5 random sampling without replacement for each amplicon count) was passed to BAMClipper and Cutadapt for primer removal of the same 1 M read pairs. For sequencing read length, the same 1 M read pairs were further trimmed to pairs of 100, 125, 150, 175, 200, 225 or 250 nt sequencing reads using Seqtk (https://github.com/lh3/seqtk) (trimfq -q 1). For sequencing read count, 1000 to 1000000 sequencing read pairs (2 × 275 nt; 5 random sampling without replacement for each read count) were randomly sampled.

### Reference sequences

Human reference genome sequence: GRCh37/hg19, *BRCA1*: NM_007294.3, *BRCA2*: NM_000059.3. Variants were described according to the recommendations of Human Genome Variation Society (HGVS)^19^. Variant descriptions were checked by Mutalyzer Name Checker^19^.

### Ethics statement

The study involving the breast and ovarian cancer panel was approved by the Institutional Review Board of the University of Hong Kong/Hospital Authority West Cluster and other contributing hospitals in Hong Kong. The study involving the myeloid neoplasm panel was approved by the Research Ethics Committee of Hong Kong Sanatorium & Hospital (reference number: REC-2015-02). All participants gave written informed consent; with the exception that informed consent is not needed for the use of a pre-existing peripheral blood sample from a de-identified healthy adult and 8 de-identified mutation control samples. All methods were performed in accordance with the relevant guidelines and regulations of the institution.

## Electronic supplementary material

Supplementary Information
