# Supplementary material for: BAMClipper: removing primers from alignments to minimize false-negative mutations in amplicon next-generation sequencing
Source: Sci Rep. 2017 May 8;7:1567. doi: 10.1038/s41598-017-01703-6 (PMC5431517; doi:10.1038/s41598-017-01703-6)
Supplement: Supplementary file 1 — Supplementary Information [file 41598_2017_1703_MOESM1_ESM.pdf]

Supplementary Information for:

BAMClipper: removing primers from alignments to minimize false-negative mutations in amplicon next-generation sequencing

Chun Hang Au[1], Dona N. Ho[1], Ava Kwong[2,3,4], Tsun Leung Chan[1], Edmond S. K. Ma[1*]

Supplementary Figure S1. A *BRCA1* deletion escaped from variant calling when primers were trimmed before mapping by BowTie 2.
Supplementary Figure S2. Additional simulated indels detected by 3 approaches of primer handling.
Supplementary Figure S3. BAMClipper showed improved computing performance and maintained high effectiveness of primer removal.
Supplementary Table S1. Comparison of primer handling approaches in detecting known variants from the myeloid neoplasm gene panel.
Supplementary Note. Source code of BAMClipper and Cutadapt pipelines used in benchmarking.

Supplementary Figure S1. A *BRCA1* deletion escaped from variant calling when primers were trimmed before mapping by BowTie 2. NGS read alignments of *BRCA1* c.1620_1636del allele from three primer handling approaches are shown in conjunction with the amplicon design and reference genome sequence. Individual forward and reverse sequencing reads after any soft-clipping were represented by red and purple horizontal lines, respectively. The expected deletion event (black box) was present in the alignments from approaches 2 and 3 only.

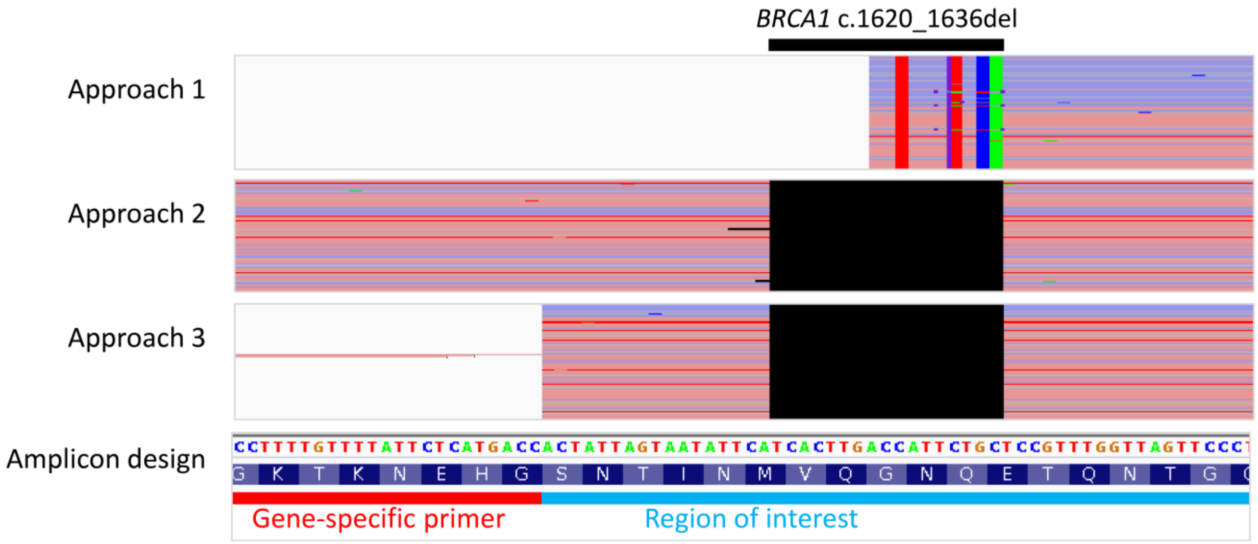

Supplementary Figure S2. Additional simulated indels detected by 3 approaches of primer handling.

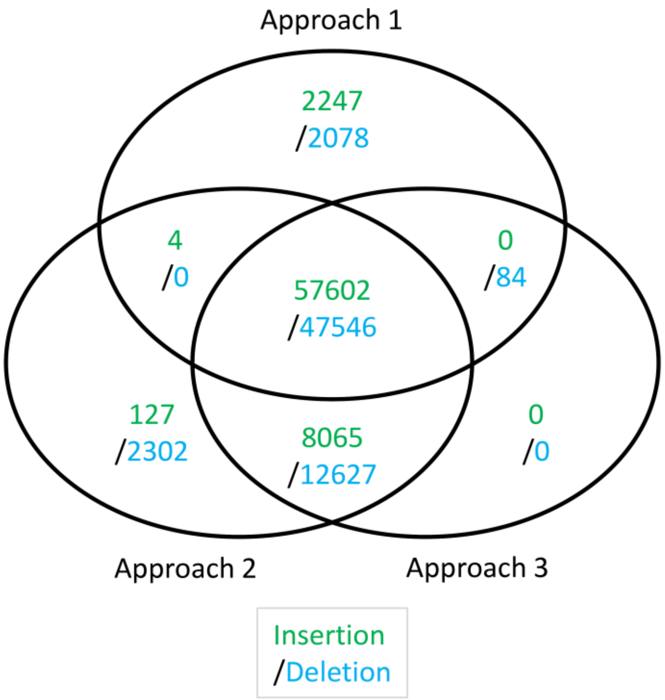

Supplementary Figure S3. BAMClipper showed improved computing performance and maintained high effectiveness of primer removal. Computing time of 1 million read pairs for (A) increasing number of amplicons and (B) increasing length of sequencing read. (C) Computing time for increasing number of sequencing read pairs. A linear regression line is also shown for each method. (A-C) Computing time of BAMClipper or Cutadapt alone was shown as "BAMClipper only" and "Cutadapt only", respectively. Overall computing time from FASTQ to primer trimmed/clipped BAM files (including read alignment time) was shown as "BAMClipper overall" and "Cutadapt overall". (D) Mean decrease of alignment depth of region of interest or gene-specific primer sites of individual amplicons. Error bars represent 1 standard deviation.

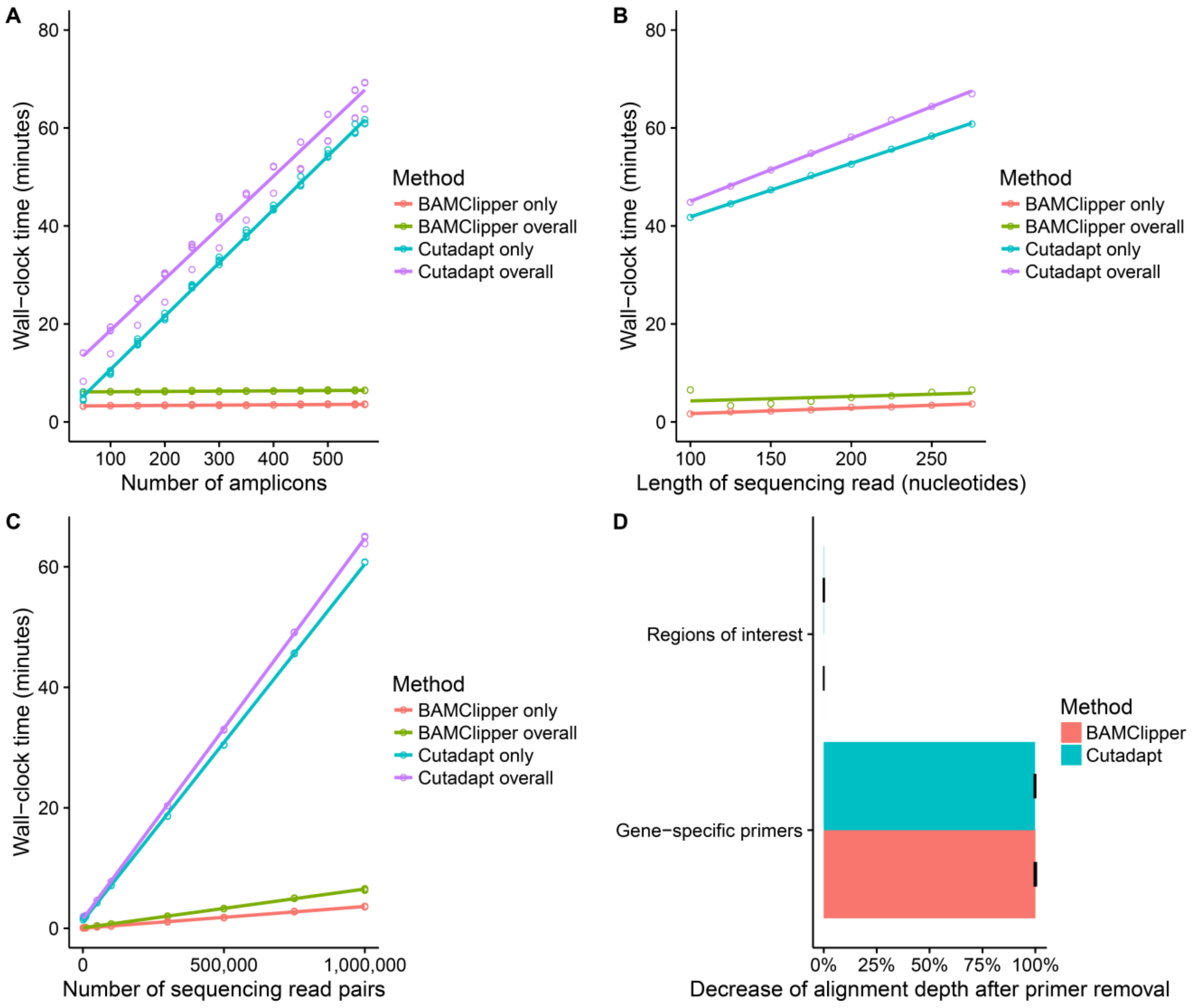

Supplementary Table S1. Comparison of primer handling approaches in detecting known variants from the myeloid neoplasm gene panel.

Sample	Mutation^	Mutation type	Approach 1 (Cutadapt)	Approach 2	Approach 3 (BAMClipper)
1	*JAK2* c.1849G>T	SNV	Detected	Detected	Detected
2	*KIT* c.2447A>T	SNV	Detected	Detected	Detected
3	*MYD88* c.794T>C	SNV	Detected	Detected	Detected
4	*TP53* c.916C>T	SNV	Detected	Detected	Detected
5	*CALR* c.1154_1155insTTGTC	Insertion (5 nt)	Detected	Detected	Detected
6	*CALR* c.1124_1142del	Deletion (19 nt)	Detected	Detected	Detected
7	*CALR* c.1103_1136del	Deletion (34 nt)	Detected	Detected	Detected
8	*CALR* c.1099_1150del (hotspot type 1)	Deletion (52 nt)	Not detected due to soft-clipping	Detected	Detected

^Reference sequences: *CALR*: NM_004343.3, *JAK2*: NM_004972.3, *KIT*: NM_000222.2, *MYD88*: NM_002468.4, *TP53*: NM_000546.4

Supplementary Note. Source code of BAMClipper and Cutadapt pipelines used in benchmarking.

BAMClipper pipeline:

```
#!/bin/bash
# BAMClipper pipeline: FASTQ > BWA-MEM > BAMClipper
# ./bamclipper_pipeline_cray.sh samplename_R1.fastq.gz samplename_R2.fastq.gz
samplename trusight_myeloid.bedpe 16
#
R1=$1
R2=$2
NAME=$3
BEDPE=$4
NUMTHREAD=$5

STARTOVERALL=$(date +%s);
####

# bwa & index
START=$(date +%s);
bwa mem -R '@RG\tID:'$NAME'\tSM:'$NAME -M -t $NUMTHREAD -L 5 ucsc.hg19.fasta $R1 $R2
| samtools view -bS - | samtools sort -@ $NUMTHREAD -m 1536M > ${NAME}.bam && samtools
index ${NAME}.bam
END=$(date +%s);
echo "### bwa" $((END-START))

# bamclipper
START=$(date +%s);
./bamclipper.sh -b ${NAME}.bam -p $BEDPE -n $NUMTHREAD
END=$(date +%s);
echo "### bamclipper" $((END-START))

####
ENDOVERALL=$(date +%s);
echo "#### overall" $((ENDOVERALL-STARTOVERALL))
```

Cutadapt pipeline:

```
#!/bin/bash
# cutadapt pipeline: FASTQ > split > parallel cutadapt > merge > BWA-MEM
# ./cutadapt_pipeline_cray.sh samplename_R1.fastq.gz samplename_R2.fastq.gz
samplename cutadapt.R1-FR.opts cutadapt.R2-FR.opts 16
#
R1=$1
R2=$2
NAME=$3
CUTADAPTR1=$4
CUTADAPTR2=$5
NUMTHREAD=$6

STARTOVERALL=$(date +%s);
####

# zcat
START=$(date +%s);
zcat $R1 > ${NAME}_R1.fastq
zcat $R2 > ${NAME}_R2.fastq
END=$(date +%s);
echo "### zcat" $((END-START))

# split fastq (fastq-splitter is available from
http://kirill-kryukov.com/study/tools/fastq-splitter/)
START=$(date +%s);
perl fastq-splitter.pl --n-parts $NUMTHREAD ${NAME}_R1.fastq
perl fastq-splitter.pl --n-parts $NUMTHREAD ${NAME}_R2.fastq
END=$(date +%s);
echo "### split" $((END-START))

# parallel cutadapt
START=$(date +%s);
for i in $(seq -w 1 $NUMTHREAD); do
    CUTADAPT_OPTS_R1=`cat $CUTADAPTR1`
    CUTADAPT_OPTS_R2=`cat $CUTADAPTR2`
    echo "cutadapt $CUTADAPT_OPTS_R1 -m 0 -e 0.1 -n 2 ${NAME}_R1.part-$i.fastq -o
${NAME}_R1.part-$i.trimmed.fastq >/dev/null; cutadapt $CUTADAPT_OPTS_R2 -m 0 -e 0.1
```

```
-n 2 ${NAME}_R2.part-$i.fastq -o ${NAME}_R2.part-$i.trimmed.fastq >/dev/null" >>
$NAME.cutadapt.commands.list
done
parallel --joblog $NAME.cutadapt.parallel.log -j $NUMTHREAD <
$NAME.cutadapt.commands.list
END=$(date +%s);
echo "### cutadapt" $((END-START))

# merge
START=$(date +%s);
cat ${NAME}_R1.part-*.trimmed.fastq > ${NAME}_R1.trimmed.fastq
cat ${NAME}_R2.part-*.trimmed.fastq > ${NAME}_R2.trimmed.fastq
END=$(date +%s);
echo "### merge" $((END-START))

# bwa & index
START=$(date +%s);
bwa mem -R '@RG\tID:'$NAME'\tSM:'$NAME -M -t $NUMTHREAD -L 5 ucsc.hg19.fasta
${NAME}_R1.trimmed.fastq ${NAME}_R2.trimmed.fastq | samtools view -bS - | samtools
sort -@ $NUMTHREAD -m 1536M > ${NAME}.bam && samtools index ${NAME}.bam
END=$(date +%s);
echo "### bwa" $((END-START))


####
ENDOVERALL=$(date +%s);
echo "#### overall" $((ENDOVERALL-STARTOVERALL))
```
